# Increased CD4^+^ T cell count is associated with lower anal human papillomavirus prevalence among HIV-positive male cohort in Taizhou, China: a cross-sectional study

**DOI:** 10.1186/s12879-022-07251-3

**Published:** 2022-03-14

**Authors:** Jing Zhang, Xiaoxiao Chen, Yiwen Ye, Weiwei Shen, Xiaohong Ye, Yajun Lin, Zhebin Lin, Shigang Tan, Meiyang Gao, Yingying Ding, Haijiang Lin, Youyi Wang, Na He, Xing Liu

**Affiliations:** 1 Department of Epidemiology, School of Public Health, and the Key Laboratory of Public Health Safety of Ministry of Education, Fudan University, Shanghai, China; 2 Taizhou City Center for Disease Control and Prevention, Taizhou, Zhejiang China; 3 Linhai District Center for Disease Control and Prevention, Taizhou, Zhejiang China; 4 Sanmen District Center for Disease Control and Prevention, Taizhou, Zhejiang China; 5 Wenling District Center for Disease Control and Prevention, Taizhou, Zhejiang China; 6 Huangyan District Center for Disease Control and Prevention, Taizhou, Zhejiang China

**Keywords:** Anal HPV infection, CD4, Antiretroviral therapy, HIV/AIDS, Male

## Abstract

**Objectives:**

This study aims to investigate the association between CD4^+^ T cell count and combined antiretroviral therapy (cART) with the prevalence of anal human papillomavirus (HPV) infection among HIV-positive male cohort in China.

**Methods:**

A survey was conducted in men from a HIV cohort in Taizhou, China between 2016 and 2019. A face-to-face questionnaire interview was administered, and an anal-canal swab was collected for HPV genotyping.

**Results:**

A total of 766 HIV-positive men were recruited. The HPV prevalence was lower among those with increased CD4^+^ T cell count than those with decreased or unchanged (46.5 vs. 56.6%, p = 0.033) from baseline. In multivariable models, having the current CD4^+^ T cell count of 350–499 cells/µL (aOR 0.28, 95% CI 0.13–0.64), and of ≥ 500 cells/µL (aOR 0.26, 95% CI 0.11–0.60) were associated with lower prevalence of any type HPV infection compared with those with < 200 cells/µL. Having taken NVP + 3TC + AZT was inversely associated with any high-risk (HR)-HPV (aOR 0.47, 95% CI 0.25–0.90) and any low-risk (LR)-HPV infection (aOR 0.40, 95% CI 0.18–0.88), compared with those taking EFV + 3TC + TDF.

**Conclusions:**

Increased CD4^+^ T cell count at follow-up was significantly associated with lower prevalence of anal HPV infection. Inverse associations between NVP + 3TC + AZT and HR-HPV or LR-HPV infecton were observed.

## Introduction

Human papillomavirus (HPV) infection is the most prevalent sexually transmitted infection (STI) in the world [1, 2]. Persistent high risk (HR)-HPV infection is the cause of almost all cervical cancer, and is a crucial oncogenic driver in other cancers including anogenital and head and neck cancers [3, 4]. Studies have shown that HIV infection is associated with higher HPV prevalence and incidence [5–8]. The prevalence of anal HPV infection is higher in HIV-positive populations than in HIV-negatives, especially among HIV-positive men who have sex with men (MSM) across the world, ranging from 82.7% to 95.0% in recent reports [9–14]. Shared transmission route for both viruses may explain part of the high HPV prevalence among HIV population, however, to what extent the impaired immune function plays a role in HPV infection remains a research question. Studies of the impact of CD4^+^ T cell count, as the commonly used proxy for immune function, on the natural history of HPV infection have generated inconclusive results. While some studies showed that increased CD4^+^ T cell count is associated with lower risk of HPV infection among HIV-positives [15–17], others had observed null associations [5, 6, 18]. On the other hand, cART is reported to affect the anal HPV prevalence, anal intraepithelial neoplasia, and anal cancer. A recent worldwide meta-analysis evidenced that effective cART use is associated with a decreased prevalence of anal HR-HPV infection in HIV- positives [19]. In addition, a longitudinal study observed a significant beneficial effect from cART against acquisition of anal HR-HPV infection in HIV positive MSM [20], while negative or null associations were also reported [21, 22]. Furthermore, few studies have looked into associations between specific cART drugs or treatments with HPV infection, although there have been hypotheses that protease inhibitors may have off-target effect on infections including HPV[23].

Development of strategies for the prevention and treatment of HPV-related cancers in the anogenital tract requires a better understanding of the risk factors and natural history of persistent HPV infection, especially given that HPV vaccine has not been widely available in China. A cross-sectional study was conducted in male participants from an ongoing HIV cohort to investigate HPV prevalence from anal canal swab samples. We hypothesized an inverse association between CD4^+^ T cell improvement and anal HPV infection, and hypothesized that cART treatments were correlated with HPV infection in the HIV-positive male population.

## Materials and methods

### Study design and subject

Participants in this study were those HIV infected men registered in and routinely followed-up by Taizhou City Center for Disease Control and Prevention (CDC) and four district-level CDCs in Taizhou, Zhejiang Province. A survey for anal HPV infection was conducted between 2016 to 2019. The eligible criteria included: (1) aged 18 years or older; (2) had baseline or current test result for CD4^+^ T cell count when the survey was conducted; (3) willing to have an anal swab sample collected. There were 1,304 HIV infected men being followed-up from the study sites, and 776 (58.7%) were willing to participate in this survey. Ten of them had neither the baseline nor the current CD4^+^ T cell test results and thus were excluded. A total of 766 participants were included in this analysis. The study was approved by the Institutional Review Board of School of Public Healh, Fudan University, and all the participants provided written informed consent.

### Data collection

Most of the participants’ socio-demographic characteristics, HIV-related data including cART treatments and multiple CD4^+^ T cell counts were obtained from the Chinese HIV/AIDS Comprehensive Information Management System. For this survey, participants were face-to-face interviewed by trained local public health staff during the visit, and a questionnaire was used to obtain epidemiological data. Overall, the following information was obtained: age; level of education; marital status with women; sexual orientation; self-reported history of STIs (including gonorrhea, syphilis, chlamydia, condyloma acuminata and genital herpes); number of lifetime sex partners; condom use in the last six months; smoking status and alcohol consumption. Ever smoked was defined as having ever smoked more than 100 cigarettes lifetime [24]. Frequency of alcohol consumption in the past year was recorded.

### cART treatment and CD4^+^ T cell counts

Of all 766 participants, 741 participants were under cART based on the recommendation from the manual of national free antiretroviral treatment program for HIV/AIDS in China [25]. Participants received one combination of two nucleoside reverse transcriptase inhibitors (NRTIs) and either a protease inhibitors (PIs) or a non-nucleoside reverse transcriptase inhibitors (NNRTIs), or a NRTIs/PI combination regimen plus another antiretroviral drug. In this study, participants mainly received the combination of two NRTIs plus one NNRTI. cART treatments were classified as four groups in the final analysis: EFV + 3TC + TDF (a combination of Efavirenz, Lamivudine and Tenofovir); EFV + 3TC + AZT (a combination of Efavirenz, Lamivudine and Zidovudine); NVP + 3TC + AZT (a combination of Nevirapine, Lamivudine and Zidovudine); and all other combined with limited number of people using them.

Baseline CD4^+^ T cell count was measured at the time of HIV diagnosis, and current CD4^+^ T cell count was measured at the follow-up which was most close to the time of HPV test, representing the baseline and recent immune status of the participants, respectively. The median time interval was 2.3 years (interquartile range [IQR], 0.7–5.0 years) between these two CD4 measurement. The change of CD4^+^ T cell counts was also calculated by current CD4 minus baseline CD4. If the result was positive, we defined those participants as having “increased CD4^+^ T cell counts”; and if the result was negative or zero, we defined those participants as having “decreased or unchanged CD4^+^ T cell counts”. Baseline and the current CD4^+^ T cell count were recorded from the system, which had been measured by local CDCs using flow cytometry (Becton, Dickinson and Company, USA) according to the manufacturer’s protocol.

### Sample collection and laboratory tests

For each participant, an anal canal swab was collected by the trained staff. A nylon flocked swab was inserted into the anal canal for 2–3 cm in depth, rotated with gentle pressure against the canal wall while being slowly moved out. The whole process of swab sample collection was required to be no less than 2 min to gain enough cells. Then the swab was kept in a tube with 3 ml liquid medium using PBS as the main component to preserve the cells, and stored at − 20 °C waiting for HPV DNA extraction. HPV genotyping was performed using the HPV GenoArray Test Kit (Hybribio Ltd., Guangdong, China). The test kit detects 21 HPV genotypes using a L1 consensus primer-based polymerase chain reaction assay by the flow-through hybridisation technique with a TC-96/G/H6 HPV DNA Amplification Analyzer and an HMM-2 fast nucleic acid molecule hybridisation instrument. Among the 21 HPV genotypes, HPV 16, 18, 31, 33, 35, 39, 45, 51, 52, 56, 58, 59 are carcinogenic types, HPV68 is a probably carcinogenic type, and HPV53 and 66 are possibly carcinogenic types, defined by the International Agency for Research on Cancer (IARC) [26]. Here we define them all as the high-risk genotypes of HPV. And the other six are LR-HPV, including 6, 11, 42, 43, 44 and 81 [27]. Biotin control, internal control, positive control and negative control from the kit were used in each laboratory test as quality control, and only when both of the biotin control and internal control showed coloration, the test was defined as valid and the genotyping results could be read and reported.

### Statistical analyses

Chi-Square test and Fisher exact test were used to compare the distribution of categorical variables where appropriate. Unconditional univariable and multivariable logistic regression models were used to estimate the odds ratios (OR) and 95% confidence intervals (CI) in determining the association between the prevalence of HPV infection and CD4^+^ T cell count or cART. Multivariable logistic regression models were adjusted for age (18–24, 25–34, 35–44, 45–54, 55–82 years), level of education (illiterate or primary school, middle school, high school, college or above), marital status (never married, currently married, widowed/divorced), smoking amount (pack year), alcohol consumption (drank≤ once/month, drank > once/month), sexual orientation (hetero-sexual, homo-sexual, bi-sexual or uncertain), self-reported history of STIs (no or yes), life-time multiple sex partners (no or yes), condom use in the last six months(no or yes). A two-sided p-value < 0.05 was considered as statistically significant. All statistical analyses were performed using SAS 9.4 statistical software (SAS Institute Inc., Cary, NC).

## Results

A total of 766 HIV-positive male participants with median age of 46.9 years (range, 18.0–82.0 years) were included in this analysis. 741 participants were under cART (96.7%). Distribution of the socio-demographic and HIV-related characteristics, as well as the prevalence of HPV infection are shown in Table 1. The majority of the study population had a level of education of middle school or lower. One third of the participants were never married; 54.0% reported their sexual orientation as heterosexual, 35.0% homosexual, and 11.0% bisexual or uncertain. 26.5% of the participants self-reported a history of STIs. The median baseline CD4^+^ T cell count was 264 cells/µL (interquartile range [IQR], 152–344 cells/µL), and the median current CD4^+^ T cell count was 446 cells/µL (IQR, 282–588 cells/µL). About half of the participants were having EFV + 3TC + TDF for cART treatment, 25.1% were EFV + 3TC + AZT, 16.7% were NVP + 3TC + AZT, and all others combined accounted for 8.8% (Table 1).

**Table 1:** Socio-demographic characteristics and prevalence of anal HPV infection among HIV-positive males in Taizhou, China

	Overall		Any HPV positive		Any HR-HPV positive		Any LR-HPV positive	
	Number	Proportion %	Number	Prevalence %	Number	Prevalence %	Number	Prevalence %
Total	766	100	373	48.7	309	40.3	173	22.6
Age (years)				P = 0.009		P < 0.001		P < 0.001
18–24	33	4.4	19	57.6	18	54.6	8	24.2
25–34	150	20.1	84	56.0	71	47.3	52	34.7
35–44	152	20.3	70	46.1	59	38.8	32	21.1
45–54	202	27.0	105	52.0	91	45.1	46	22.8
55–82	211	28.2	82	38.9	58	27.5	29	13.7
Missing	18							
Level of education				P = 0.019		P < 0.001		P < 0.001
Illiterate or primary school	211	28.2	83	39.3	58	27.5	25	11.9
Middle school	315	42.0	159	50.5	137	43.5	83	26.4
High school	140	18.7	71	50.7	63	45.0	34	24.3
College or above	83	11.1	47	56.6	39	47.0	26	31.3
Missing	17							
Marital status				P = 0.002		P < 0.001		P = 0.002
Never married	250	33.4	142	56.8	122	48.8	75	30.0
Currently married	365	48.7	154	42.2	117	32.1	65	17.8
Widowed/divorced	134	17.9	64	47.8	58	43.3	28	20.9
Missing	17							
Sexual orientation				P < 0.001		P < 0.001		P < 0.001
Hetero-sexual	406	54.0	160	39.4	112	27.6	50	12.3
Homo-sexual	263	35.0	157	59.7	146	55.5	97	36.9
Bi-sexual or uncertain	83	11.0	52	62.7	49	59.0	24	28.9
Missing	14							
Self-reported history of STIs				P = 0.048		P = 0.007		P = 0.009
No	503	73.5	232	46.1	187	37.2	102	20.3
Yes	181	26.5	99	54.7	88	48.6	54	29.8
Missing	82							
Multiple sex partners^a^				P = 0.770		P = 0.753		P = 0.090
No	49	6.5	25	51.0	21	42.9	16	32.7
Yes	700	93.5	342	48.9	284	40.6	155	22.1
Missing	17							
Condom use^a^				P = 0.027		P = 0.006		P = 0.062
Yes	8	1.0	7	87.5	7	87.5	4	50.0
No	758	99.0	366	48.3	302	39.8	169	22.3
Missing	0							
Baseline CD4^+^ T (cells/µL)				P = 0.883		P = 0.947		P = 0.860
< 200	265	35.0	127	47.9	103	38.9	56	21.1
200–349	310	41.0	153	49.4	128	41.3	68	21.9
350–499	120	15.9	56	46.7	48	40.0	30	25.0
≥ 500	61	8.1	32	52.5	25	41.0	13	21.3
Missing	10							
Current CD4^+^ T (cells/µL)				P = 0.091		P = 0.086		P = 0.851
< 200	88	12.1	51	58.0	47	53.4	21	23.9
200–349	192	26.5	99	51.6	74	38.5	44	22.9
350–499	175	24.2	75	42.9	69	39.4	36	20.6
≥ 500	270	37.2	126	46.7	106	39.3	65	24.1
Missing	41							
Change of CD4^+^ T^b^ (cells/µL)				P = 0.033		P = 0.093		P = 0.578
Decreased or unchanged	136	19.0	77	56.6	64	47.1	28	20.6
Increased	579	81.0	269	46.5	227	39.2	132	22.8
Missing	51							
cART				P = 0.417		P = 0.298		P = 0.157
EFV + 3TC + TDF	366	49.4	186	50.8	151	41.3	82	22.4
EFV + 3TC + AZT	186	25.1	92	49.5	80	43.0	46	24.7
NVP + 3TC + AZT	124	16.7	54	43.6	41	33.1	19	15.3
Others combined	65	8.8	28	43.1	24	36.9	18	27.7
Missing	25							
Duration of cART (years)				P = 0.017		P = 0.025		P = 0.201
< 2	300	40.5	163	54.3	135	45.0	76	25.3
2– < 5	268	36.2	126	47.0	105	39.2	57	21.3
≥ 5	173	23.3	71	41.0	56	32.4	32	18.5
Missing	25							

*HR-HPV* high-risk HPV, *LR-HPV* low-risk HPV, *STIs* sexually transmitted infections, *cART* combined antiretroviral therapy (741 participants were under cART), *EFV* Efavirenz, *3TC* Lamivudine, *TDF* Tenofovir, *AZT* Zidovudine, *NVP* Nevirapine

^a^Life-time multiple sex partners; condom use in the last 6 months

^b^The change of CD4 + T cell counts was calculated by current CD4 minus baseline CD4. If the result was positive, it was defined as “increased CD4 + T cell counts”; and if the result was negative or zero, it was defined as “decreased or unchanged CD4 + T cell counts”

**Fig. 1:**
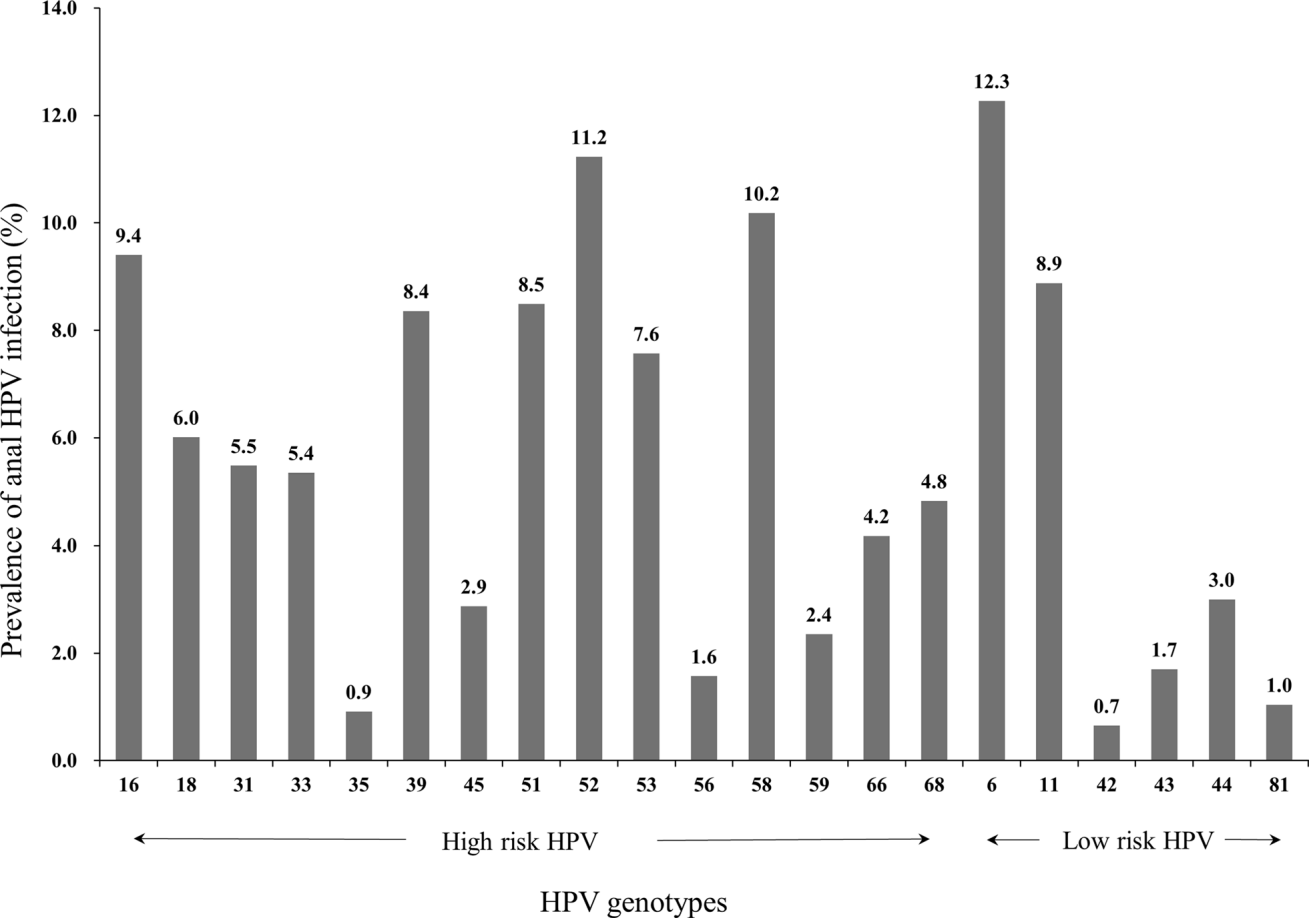
The prevalence of each genotype of anal HPV infection among HIV-positive males in Taizhou, China. There are 16.7% participants infected with only one genotype of HPV, and 29.0% participants infected with two or more than two genotypes (multiple infections). The prevalence adds up to more than 100% because of multiple infection

The prevalence of each genotype of anal HPV infection is shown in Fig. 1, with HPV-52 (11.2%), HPV-58 (10.2%) and HPV-16 (9.4%) being the top three for HR-HPV, and HPV-6 (12.3%), HPV-11 (8.9%) and HPV-44 (3.0%) being the top three for LR-HPV. There are 16.7% participants infected with only one genotype of HPV, and 29.0% participants infected with two or more than two genotypes (multiple infections). Overall, the prevalence of anal HPV infection was 48.7% for any HPV type, 40.3% for any HR-HPV, and 22.6% for any LR-HPV (Table 1). The prevalence of anal HPV infection decreased significantly with the increase of age, and was higher among those with higher level of education. The prevalence of any type of HPV infection was 39.4% for heterosexual, lower than the 59.7% for homosexual and 62.7% for bisexual or uncertain (p < 0.001). The HPV prevalence was lower among those with increased CD4^+^ T cell count than those with decreased or unchanged (46.5 vs. 56.6%, p = 0.033) comparing current to baseline tests. The prevalence of HPV was lower among those with longer duration on cART (p = 0.017). Similar differences were observed for HR-HPV and LR-HPV prevalence for most of the above-mentioned characteristics (Table 1).

Univariable (Table 2) and multivariable (Table 3) logistic regression models were conducted to examine the associations between CD4^+^ T cell counts, cART treatments, duration of cART and other characteristics with prevalence of HPV. In multivariable models, after adjusting for covariates, having the current CD4^+^ T cell count of 350–499 cells/µL (aOR 0.28, 95% CI 0.13–0.64), and of ≥ 500 cells/µL (aOR 0.26, 95% CI 0.11–0.60) were inversely associated with prevalence of any type HPV infection compared with those of < 200 cells/µL. Similar significant associations were observed for any HR-HPV infection, but not LR-HPV. Having taken NVP + 3TC + AZT compared with those taking EFV + 3TC + TDF was inversely associated with any HR-HPV infection (aOR 0.47, 95% CI 0.25–0.90), and inversely associated with any LR-HPV infection (aOR 0.40, 95% CI 0.18–0.88), but not any HPV infection. The association between duration of cART and any type of HPV infection had not been observed. The longer duration on cART was associated with lower HPV prevalence in univariable model, while the association was null in multivariable model (Tables 2 and 3).

**Table 2:** Univariable logistic regression for associations between socio-demographics, CD4 and cART with anal HPV infection among HIV-positive males in Taizhou, China

	Any HPV		Any HR-HPV		Any LR-HPV	
	OR (95% CI)	P-value	OR (95% CI)	P-value	OR (95% CI)	P-value
Age(years)						
18–24	1.00		1.00		1.00	
25–34	0.94 (0.44, 2.01)	0.869	0.75 (0.35, 1.60)	0.454	1.66 (0.70, 3.94)	0.251
35–44	0.63 (0.29, 1.35)	0.232	0.53 (0.25, 1.13)	0.100	0.83 (0.34, 2.02)	0.687
45–54	0.80 (0.38, 1.68)	0.551	0.68 (0.33, 1.43)	0.312	0.92 (0.39, 2.18)	0.852
55–82	0.47 (0.22, 0.99)	0.046	0.32 (0.15, 0.67)	0.003	0.50 (0.21, 1.21)	0.124
Level of education						
Illiterate or primary school	1.00		1.00		1.00	
Middle school	1.57 (1.10, 2.24)	0.012	2.03 (1.40, 2.96)	< 0.001	2.66 (1.64, 4.33)	< 0.001
High school	1.59 (1.03, 2.44)	0.036	2.16 (1.38, 3.38)	< 0.001	2.39 (1.35, 4.22)	0.003
College or above	2.01 (1.20, 3.37)	0.008	2.34 (1.38, 3.96)	0.002	3.39 (1.82, 6.33)	< 0.001
Marital status						
Never married	1.00		1.00		1.00	
Currently married	0.56 (0.40, 0.77)	< 0.001	0.50 (0.36, 0.69)	< 0.001	0.51 (0.35, 0.74)	< 0.001
Widowed/divorced	0.70 (0.46, 1.06)	0.091	0.80 (0.53, 1.22)	0.302	0.62 (0.38, 1.01)	0.056
Smoking amount (packyear)						
0	1.00		1.00		1.00	
0–20	0.85 (0.59, 1.25)	0.413	0.71 (0.48, 1.05)	0.088	0.82 (0.52, 1.29)	0.384
> 20	0.44 (0.28, 0.68)	< 0.001	0.44 (0.28, 0.70)	< 0.001	0.44 (0.24, 0.80)	0.007
Alcohol consumption						
Drank≤once/month	1.00		1.00		1.00	
Drank > once/month	0.69 (0.50, 0.95)	0.022	0.59 (0.42, 0.82)	0.002	0.74 (0.49, 1.10)	0.132
Sexual orientation						
Hetero-sexual	1.00		1.00		1.00	
Homo-sexual	2.28 (1.66, 3.13)	< 0.001	3.28 (2.36, 4.54)	< 0.001	4.16 (2.82, 6.13)	< 0.001
Bi-sexual or uncertain	2.58 (1.58, 4.20)	< 0.001	3.78 (2.32, 6.17)	< 0.001	2.90 (1.66, 5.07)	< 0.001
Self-reported history of STIs						
No	1.00		1.00		1.00	
Yes	1.41 (1.00, 1.98)	0.048	1.60 (1.14, 2.25)	0.007	1.67 (1.14, 2.46)	0.009
Multiple sex partners^a^						
No	1.00		1.00		1.00	
Yes	0.92 (0.51, 1.64)	0.770	0.91 (0.51, 1.64)	0.753	0.59 (0.32, 1.09)	0.093
Condom use^a^						
Yes	1.00		1.00		1.00	
No	0.13 (0.02, 1.09)	0.060	0.10 (0.01, 0.77)	0.028	0.29 (0.07, 1.16)	0.080
Baseline CD4^+^ T (cells/µL)						
< 200	1.00		1.00		1.00	
200–349	1.06 (0.76, 1.47)	0.732	1.11 (0.79, 1.55)	0.555	1.05 (0.70, 1.56)	0.815
350–499	0.95 (0.62, 1.47)	0.819	1.05 (0.68, 1.63)	0.833	1.24 (0.75, 2.07)	0.399
≥ 500	1.20 (0.69, 2.09)	0.523	1.09 (0.62, 1.93)	0.760	1.01 (0.51, 2.00)	0.975
Current CD4^+^ T (cells/µL)						
< 200	1.00		1.00		1.00	
200–349	0.77 (0.46, 1.29)	0.320	0.55 (0.33, 0.91)	0.020	0.95 (0.52, 1.72)	0.862
350–499	0.54 (0.32, 0.91)	0.021	0.57 (0.34, 0.95)	0.032	0.83 (0.45, 1.52)	0.541
≥ 500	0.64 (0.39, 1.03)	0.067	0.56 (0.35, 0.92)	0.020	1.01 (0.58, 1.78)	0.968
Change of CD4^+^ T (cells/µL)^b^						
Decreased or unchanged	1.00		1.00		1.00	
Increased	0.67 (0.46, 0.97)	0.034	0.73 (0.50, 1.06)	0.094	1.14 (0.72, 1.80)	0.578
cART						
EFV + 3TC + TDF	1.00		1.00		1.00	
EFV + 3TC + AZT	0.95 (0.66, 1.35)	0.763	1.08 (0.75, 1.54)	0.693	1.14 (0.75, 1.72)	0.540
NVP + 3TC + AZT	0.75 (0.50, 1.12)	0.162	0.70 (0.46, 1.08)	0.107	0.63 (0.36, 1.08)	0.094
Others combined	0.73 (0.43, 1.25)	0.251	0.83 (0.48, 1.44)	0.512	1.33 (0.73, 2.41)	0.353
Duration of cART (years)						
< 2	1.00		1.00		1.00	
2– < 5	0.75 (0.54, 1.04)	0.082	0.79 (0.56, 1.10)	0.161	0.80 (0.54, 1.18)	0.254
≥ 5	0.58 (0.40, 0.85)	0.006	0.58 (0.40, 0.86)	0.007	0.67 (0.42, 1.06)	0.089

*cART* combined antiretroviral therapy (741 participants were under cART), *EFV* Efavirenz, *3TC* Lamivudine, *TDF* Tenofovir, *AZT* Zidovudine, *NVP* Nevirapine

^a^Life-time multiple sex partners; condom use in the last 6 months

^b^The change of CD4 + T cell counts was calculated by current CD4 minus baseline CD4. If the result was positive, it was defined as “increased CD4 + T cell counts”; and if the result was negative or zero, it was defined as “decreased or unchanged CD4 + T cell counts”

**Table 3:** Multivariable logistic regression for associations between CD4, cART and other risk factors with anal HPV infection among HIV-positive males in Taizhou, China

	Any HPV		Any HR-HPV		Any LR-HPV	
	aOR (95% CI)	P-value	aOR (95% CI)	P-value	aOR (95% CI)	P-value
Age(years)						
18–24	1.00		1.00		1.00	
25–34	1.41 (0.53, 3.76)	0.497	1.25 (0.46, 3.38)	0.662	4.29 (1.19, 15.42)	0.026
35–44	1.04 (0.36, 2.98)	0.945	0.92 (0.32, 2.69)	0.878	2.38 (0.61, 9.28)	0.212
45–54	2.02 (0.66, 6.13)	0.215	1.80 (0.58, 5.59)	0.311	4.21 (1.02, 17.40)	0.047
55–82	1.41 (0.43, 4.61)	0.567	1.24 (0.37, 4.21)	0.725	4.20 (0.91, 19.31)	0.065
Level of education						
Illiterate or primary school	1.00		1.00		1.00	
Middle school	2.01 (1.10, 3.66)	0.023	2.84 (1.49, 5.40)	0.002	3.82 (1.71, 8.52)	0.001
High school	1.46 (0.71, 2.99)	0.301	2.11 (0.99, 4.48)	0.052	1.81 (0.71, 4.60)	0.214
College or above	1.18 (0.52, 2.70)	0.692	1.55 (0.66, 3.64)	0.315	1.26 (0.44, 3.63)	0.670
Marital status						
Never married	1.00		1.00		1.00	
Currently married	0.55 (0.31, 0.98)	0.042	0.47 (0.26, 0.86)	0.014	0.65 (0.33, 1.28)	0.211
Widowed/divorced	0.67 (0.32, 1.39)	0.282	0.86 (0.41, 1.81)	0.688	0.50 (0.20, 1.22)	0.125
Smoking amount (packyear)	1.00 (0.99, 1.00)	0.507	1.00 (0.99, 1.00)	0.514	1.00 (0.99, 1.00)	0.451
Alcohol consumption						
Drank≤once/month	1.00		1.00		1.00	
Drank > once/month	0.75 (0.46, 1.22)	0.244	0.53 (0.31, 0.89)	0.016	0.92 (0.50, 1.70)	0.801
Sexual orientation						
Hetero-sexual	1.00		1.00		1.00	
Homo-sexual	2.12 (1.32, 3.41)	0.002	2.41 (1.48, 3.92)	< 0.001	3.54 (1.97, 6.35)	< 0.001
Bi-sexual or uncertain	2.47 (1.25, 4.90)	0.010	1.98 (0.99, 3.97)	0.053	2.29 (1.00, 5.24)	0.049
Self-reported history of STIs						
No	1.00		1.00		1.00	
Yes	1.22 (0.78, 1.92)	0.378	1.29 (0.81, 2.05)	0.287	1.49 (0.88, 2.52)	0.140
Multiple sex partners^a^						
No	1.00		1.00		1.00	
Yes	0.84 (0.37, 1.92)	0.684	0.95 (0.40, 2.24)	0.903	0.35 (0.14, 0.89)	0.028
Condom use ^c^						
Yes	1.00		1.00		1.00	
No	0.17 (0.02, 1.60)	0.122	0.09 (0.01, 0.86)	0.037	0.24 (0.04, 1.42)	0.116
Baseline CD4^+^ T (cells/µL)						
< 200	1.00		1.00		1.00	
200–349	1.47 (0.88, 2.45)	0.141	1.40 (0.83, 2.37)	0.211	0.92 (0.50, 1.69)	0.792
350–499	1.13 (0.55, 2.32)	0.730	0.88 (0.42, 1.85)	0.736	1.33 (0.57, 3.10)	0.509
≥ 500	2.02 (0.82, 4.97)	0.126	1.09 (0.43, 2.76)	0.849	0.91 (0.31, 2.73)	0.872
Current CD4^+^ T (cells/µL)						
< 200	1.00		1.00		1.00	
200–349	0.58 (0.28, 1.22)	0.153	0.43 (0.20, 0.91)	0.028	0.91 (0.38, 2.15)	0.829
350–499	0.28 (0.13, 0.64)	0.002	0.28 (0.12,0.64)	0.003	0.43 (0.16, 1.12)	0.085
≥ 500	0.26 (0.11, 0.60)	0.002	0.26 (0.11, 0.62)	0.002	0.54 (0.21, 1.43)	0.215
cART						
EFV + 3TC + TDF	1.00		1.00		1.00	
EFV + 3TC + AZT	0.87 (0.51, 1.48)	0.607	1.00 (0.58, 1.73)	0.999	1.02 (0.55, 1.88)	0.958
NVP + 3TC + AZT	0.72 (0.40, 1.33)	0.299	0.47 (0.25, 0.90)	0.023	0.40 (0.18, 0.88)	0.023
Others combined	0.71 (0.35, 1.47)	0.361	0.67 (0.32, 1.42)	0.295	1.05 (0.45, 2.46)	0.906
Duration of cART (years)						
< 2	1.00		1.00		1.00	
2– < 5	0.96 (0.59, 1.57)	0.869	0.78 (0.47, 1.30)	0.336	0.74 (0.41, 1.33)	0.310
≥ 5	0.74 (0.40, 1.38)	0.343	0.63 (0.33, 1.21)	0.166	0.93 (0.44, 1.98)	0.852

*aOR* adjusted odds ratio, adjusted for all variables listed in the same column

*cART* combined antiretroviral therapy (741 participants were under cART), *EFV* Efavirenz, *3TC* Lamivudine, *TDF* Tenofovir, *AZT* Zidovudine, *NVP* Nevirapine

^a^Life-time multiple sex partners; condom use in the last 6 months

## Discussion

In this study, we report a lower prevalence of any HPV and any HR-HPV infection among people with increased CD4^+^ T cell count, and suggest an inverse association of NVP + 3TC + AZT of cART with HR-HPV and LR-HPV prevalence in a HIV-positive male population in China.

Our study found that the prevalence of anal HPV infection among HIV-positive males was high, especially for any HPV (48.7%) and any HR-HPV (40.3%), after expanding the sample size from the previous cross-sectional study [28]. HIV-positive men who have sex with men still showed the highest prevalence for anal HPV infection, which was concordant with observations all over the world [9–11, 29, 30].

We found that higher CD4^+^ T cell count was inversely associated with anal HPV infection in this HIV male population. Lower HPV and HR-HPV prevalence were not only seen among the participants from the higher current CD4^+^ T cell count groups, but also among those with an increased CD4^+^ T cell count from baseline. Yet the effect of CD4^+^ T cell counts on HPV infection is inconsistent from literature. Some studies have reported that increased CD4^+^ T cell count were associated with lower risk of HPV infection [15–17, 31, 32], but others have not observed a significant association [5, 6, 18]. We added some evidence that an improved immune function represented by CD4 might help preventing or clearing HPV infection. This also emphasized the importance of timely cART use for the prevention of HPV infection in people living with HIV.

Most studies analyzing the impact of cART on HPV infection reported beneficial effect [19, 20, 33–35]. A meta-analysis concluded that cART is associated with decreased prevalence of anal HR-HPV infection in HIV population [19]. A prospective study reported that cART reduces the risk of acquiring anal HR-HPV infection in HIV positive men who have sex with men (MSM) [20]. However, negative or null associations were also reported [21, 22]. The HIV-infected people are given cART immediately after HIV diagnoses in China since 2016. We observed that the duration on cART was marginally inversely associated with HPV infection. We also explored the associations between specific cART treatments and HPV infection, and found that participants taking NVP + 3TC + AZT showed the lowest prevalence of any HR-HPV and any LR-HPV infection compared with those taking EFV + 3TC + TDF or EFV + 3TC + AZT. Taking NVP + 3TC + AZT is also associated with a greater proportion gaining CD4 increase from baseline in the study population (data not shown), suggesting a potential indirect effect from the therapy through CD4 improvement on HPV infection. Both laboratory and human studies have looked into associations of HIV drugs, especially protease inhibitors, with HPV infection and HPV related disease progression [23]. Studies found that HIV protease inhibitor lopinavir inhibited the ability of HPV16 E6 in degrading p53 expression in vitro [36], lopinavir increased expression of antiviral protein in HPV positive cervical carcinoma cells [37], and ritonavir or saquinavir inhibited cervical intraepithelial neoplasia progression in either HIV-infected or uninfected women [38]. A small single-arm trial conducted in Kenya found that lopinavir/ritonavir is effective in clearance of HPV infection and regression of cervical leasions [39]. However, an observational study in HIV-positive women in the United States did not observe significant association between lopinavir and oncogenic HPV prevalence or clearance [40]. Although in the current study we did not have enough statistical power to examine the association of lopinavir/ritonavir (only 5% participants were using it), these interesting findings deserve further investigation in both population study on disease progression and laboratory exploration for underlying mechanism.

This study has several limitations. First, sexual behavior characteristic was based on self-reported by participants and may not completely represent actual exposure. Second, the recruitment of participants was based on a convenient sample instead of a random sample. It was possible that the participants differed in behavior or knowledge related to HPV infection from those who did not participate. Third, the testing kit for HPV used in this study was capable of detecting 21 genotypes of HPV, which did not cover all genotypes. Thus the prevalence of HPV might have been under-estimated. However, the kit included the most important genotypes of HR-HPV including 16 and 18, and the most prevalent LR-HPV 6 and 11, we think the measurement of outcome is sufficient in this study. Fourth, this study only included HIV-positive males from four CDCs of Taizhou. Hence, it is necessary to be cautious to generalize the results to all HIV-positive males in China.

## Conclusion

In conclusion, we observed lower prevalence of anal HPV infection among those HIV-infected men with increased CD4, and those taking NVP + 3TC + AZT. Given the high prevalence of HPV infection among HIV-positive males, a large-scale prospective study on natural history of HPV infection should be conducted in the future to confirm the associations. Besides the impact of CD4 and specific antiretroviral treatments, other host and environmental factors on the persistent HPV infection are of interest for further investigation.

## Data Availability

The datasets generated and/or analysed during the current study are not publicly available due to the privacy but are available from the corresponding author on reasonable request.

## Abbreviations

3TC: Lamivudine
aOR: Adjusted odds ratio
AZT: Zidovudine
cART: Combined antiretroviral therapy
CD4: Cluster of differentiation 4
CDC: Center for Disease Control and Prevention
CI: Confidence interval
DNA: Deoxyribonucleic acid
EFV: Efavirenz
HIV/AIDS: Human immunodeficiency virus/acquired immunodeficiency syndrome
HPV: Human papillomavirus
HR: High risk
IQR: Interquartile range
LR: Low risk
MSM: Men who have sex with men
NNRTIs: Non-nucleoside reverse transcriptase inhibitors
NRTIs: Nucleoside reverse transcriptase inhibitors
NVP: Nevirapine
OR: Odds ratios
PBS: Phosphate Buffer Saline
PIs: Protease inhibitors
STI: Sexual transmitted infection
TDF: Tenofovir
